# The Investigation of Posttraumatic Pseudoaneurysms in Patients Treated with Nonoperative Management for Blunt Abdominal Solid Organ Injuries

**DOI:** 10.1371/journal.pone.0121078

**Published:** 2015-03-17

**Authors:** Hirotada Kittaka, Yoshiki Yagi, Ryosuke Zushi, Hiroshi Hazui, Hiroshi Akimoto

**Affiliations:** Department of Emergency, Osaka Misihima Emergency Critical Care Center, 11-1, Minami-Akutagawa-cho, Takatsuki City, Osaka Prefecture, Japan; Massachusetts General Hospital, UNITED STATES

## Abstract

**Background:**

Posttraumatic pseudoaneurysms (PAs) have been recognized as the cause of delayed hemorrhage complicated with nonoperative management (NOM), although the need for intervention in patients with small-sized PAs and the relationship between the occurrence of PAs and bed-rest has been also unclear.

**Objectives:**

The purpose of this study was to investigate the clinical history of small-sized PAs (less than 10 mm in diameter) which occurred in abdominal solid organs, and to analyze the relationship between the occurrence of PAs and early mobilization from bed.

**Methods:**

Sixty-two patients who were successfully managed with NOM were investigated. Mobilization within three days post-injury was defined as “early mobilization” and bed-rest lasting over three days was defined as “late mobilization.” A comparison of the clinical factors, including the duration of bed-rest between patients with and without PAs detected by follow-up CT was performed. Furthermore, a multiple logistic regression model analysis on the occurrence of PAs was performed.

**Results:**

PAs were detected in 7 of the 62 patients. The One patient with PAs measuring larger than 10 mm received trans-arterial embolization, and the remaining six patients with PAs smaller than 10 mm were managed conservatively. Consequently, no delayed hemorrhage occurred, and the PAs spontaneously disappeared in all of the six patients managed without intervention. The multiple regression model analysis revealed that early mobilization was not a significant factor predicting new-onset PAs.

**Conclusions:**

Small PAs can be expected to disappear spontaneously. Moreover, early mobilization is not a significant risk factor for the occurrence of PAs.

## Background

Nonoperative management (NOM) has been established as the standard therapy for hemodynamically stable patients with blunt abdominal injuries [1–5]. Recently, adjunctive trans-catheter embolization (TAE) has reduced the rate of NOM failure even in patients with risk factors such as high-grade injury (IV-V Organ Injury Scale (OIS) of American Association of Surgery for Trauma (AAST)), older age (> 55 years), disorders of consciousness, contrast blush on computed tomography (CT) and the presence of associated injuries, and thus, the indications for NOM have been expanded [1,2,6–8]. Nonetheless, although the rates of delayed hemorrhage are rare, occurring in 5–25% of splenic, 0–3.9% of hepatic and 0–9% of renal injuries, respectively, it is a life-threatening complication of NOM and often requires an emergency laparotomy [2–5,9–11].

Posttraumatic pseudoaneurysms (PAs) have been recognized as the cause of delayed hemorrhage, and the occurrence rate in patients with NOM was reported to be 2–27% [12–18]. Some of these PAs were actually detected due to symptoms that raised a suspicion of bleeding from PAs, such as hematemesis, tarry stools and hematuria, so aggressive TAE for PAs has been recommended in the past case reports [12,19–22]. However, some PAs were found incidentally, with no symptoms, by follow-up palliative CT scans, and some of these sometimes disappeared spontaneously without any interventions [13,23–26]. The natural history and cause of PAs have been unclear, and the indications for the treatment of PAs with no symptoms have not been established.

Furthermore, to prevent trauma patients with abdominal solid organ injuries from developing delayed bleeding, strict bed-rest have been recommended, but the necessary duration and intensity of immobilization is still unclear [1,2]. A recent report described that early mobilization was not related to delayed hemorrhage [3], and referring to this study, it is possible that early mobilization may not correlate with the occurrence of PAs that lead to delayed hemorrhage. To our knowledge, there has been no report describing the relationship between early mobilization and the occurrence of posttraumatic PAs.

According to past case reports about posttraumatic PAs in the solid organs [22,27–31], the minimum size of PAs requiring of intervention because of clinical symptom was 15mm in diameter, so in our institution the indication of treatment has been limited to those with more than 10mm in diameter. The purpose of this study was 1) to explore the clinical course and outcome of patients with posttraumatic PAs (less than 10mm in diameter) treated conservatively without TAE and 2) to analyze the risk factors for the occurrence of PAs to investigate the relationship between early mobilization and the occurrence rate of PAs. We hypothesized that no relationship between the period of bed-rest and the occurrences of PAs would be confirmed, and that immobilization would not prevent delayed hemorrhage.

## Methods

### Patients

**Fig 1:**
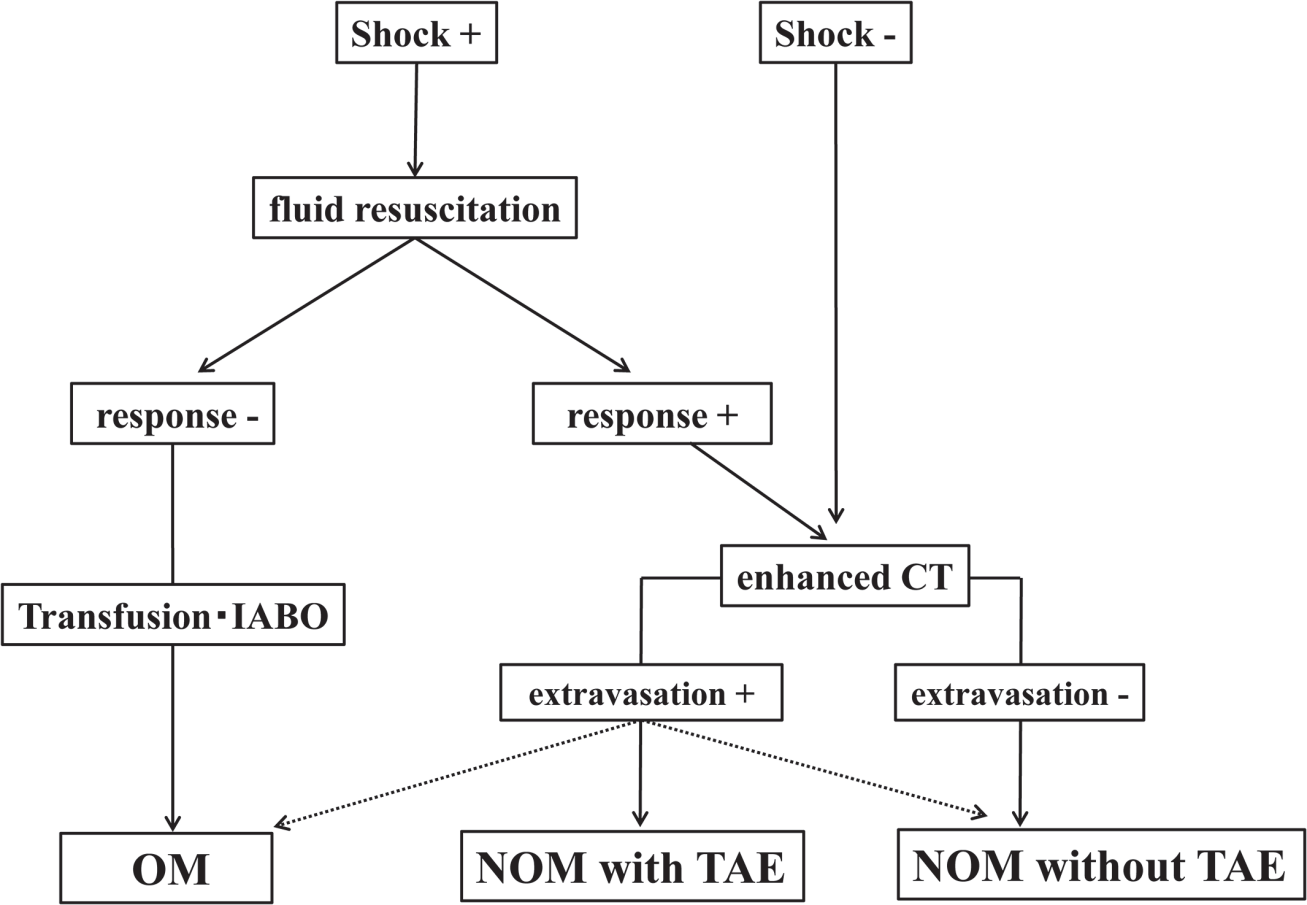
Protocol for the management of abdominal solid organ injuries.

Of the blunt trauma patients transported to Osaka Mishima emergency critical care center between 2007 and 2013, those who were treated with NOM for abdominal solid organ injuries such as those of the liver, spleen and kidneys were included in the present study. The following cases were excluded: 1) patients who were younger than 18 years of age, 2) patients in cardiopulmonary arrest on arrival, 3) patients who died in the emergency department, 4) patients forced to undergo conservative therapy due to severe head injuries with no hope of survival, 5) patients in whom PAs were detected by the initial CT scan and 6) patients who required emergency laparotomy within 24 hours post-injury due to uncontrolled hemorrhage following NOM with TAE, or detection of hollow viscous injuries, which is called “NOM failure”. In this study, delayed hemorrhage was defined as signs of intra-abdominal hemorrhage more than 48 hours post-injury, including both those that occurred during the hospital stay and those that developed after discharge. All trauma patients were managed by the standard clinical algorithm throughout the entire period of the study (Fig. 1).

### Treatment algorithm for blunt abdominal trauma patients

The treatment protocol for patients with blunt abdominal trauma in our institution is shown in Fig. 1. Patients who were hemodynamically stable at presentation or who responded to initial fluid resuscitation therapy received an imaging examination by enhanced multiple detector CT. Patients with extravasation of contrast medium from the injured organs were fundamentally considered to be indicated for NOM with TAE, however, when the vital signs worsened during the examination, operative management (OM) was selected. Patients without extravasation, who had no sources of laparotomy such as hollow viscous injuries, were managed by NOM without TAE regardless of age, disorders of consciousness, organ injury grade, or Injury Severity Score (ISS) (Fig. 1).

### Follow-up protocol for patients managed with NOM

Patients managed with NOM with or without TAE were admitted to the intensive care unit and kept on bed-rest for 24–72 hours or longer as recommended by the trauma surgeon, and during this period, the hemoglobin level was checked at least every 24 hour, and an abdominal ultrasound was repeated. A follow-up CT scan was obtained between three and seven days post-injury, based on the condition of the patient. When PAs that measured more than 10 mm were detected by follow-up CT, TAE was performed with selective catheterization for each responsible artery, while observation without interventions was selected when the diameters of the PA measured less than 10 mm. For all patients with PAs less than 10 mm, a second follow-up CT scan was performed 3–7 days after the diagnosis of PAs to check whether the diameter had increased or whether the PAs had spontaneously resolved. When the PAs were enlarged to have a diameter of more than 10 mm, palliative TAE was planned. When the PAs remained without enlargement, a follow-up CT was scanned 7–14 days after.

### Statistical analyses

The following clinical factors were correlated with the new-onset of PA after the initiation of NOM: gender, age, injured organ, grade 4 or 5 of OIS in the AAST guidelines, the existence of extravasation in the initial CT, pro-thrombin activity (PT-act), fibrinogen level, ISS, and the period of bed-rest. Patients who could be mobilized from bed within three days post-injury were defined as the “early mobilization” group and those who were kept on bed-rest for more than three days were considered to be the “late mobilization” group. The data are presented as the median (range), where appropriate. Cross tabulations using either Pearson’s chi-square tests or Fisher’s exact tests were performed to compare the clinical characteristics among the patients with and without new-onset PAs. Comparisons of continuous variables with the occurrence of PAs were performed using the Mann-Whitney U test. A multivariable logistic regression analysis was also performed to determine the independently significant factors associated with the occurrence of PAs. The values of P < 0.05 were considered to be significant. All analyses were performed using JMP 10 statistical software package (SAS Institute Inc., Cary, NC, USA).

### Ethics Statement

All procedures were approved by the Ethics Committee of Osaka Mishima Emergency Critical Center and were carried out in accordance with the Declaration of Helsinki (2004). Written informed consent was obtained from all patients enrolled in this study.

## Results

### Overall patient characteristics

During the study period, 68 patients were managed with NOM, and five patients were considered to have NOM failure because of uncontrolled bleeding in three patients managed with TAE (two required emergency laparotomy, one died without undergoing an operation) and of other abdominal organ injuries in two patients (one was pancreatic injuries, one with duodenal perforation), so the success rate of NOM was 93% (63/65). One other patient was excluded because a PA was detected by the initial CT scan, which was resolved by TAE. Therefore, as shown in Table 1, 62 patients were included in the NOM study. There were 44 males and 18 females, with a median age of 37 (18–83) years, and some of those had multiple abdominal solid organ injuries. The details of a total of the injured organs were as follows: the liver was injured in 28, the spleen in 19 and a kidney was injured in 15 cases. A total of 27 of the 62 (44%) patients had a grade 4 or 5 OIS in each organ. Shock was defined by a systolic blood pressure < 90 mmHg or a shock index (heart rate /systolic blood pressure) > 1.0 on arrival to the emergency department, and was considered to be present in 31% (19/62) of the patients. All of those shock patients were responders to the initial fluid resuscitation. Consequently, enhanced CT was performed for all 62 patients, and 19 patients (31%) were revealed to have abdominal solid organ injuries with extravasation, 16 of whom were managed by NOM with TAE, and the remaining three patients were treated by NOM without TAE. Thus, 46 patients received NOM without TAE. Nineteen of the 62 (31%) patients were able to be mobilized within 72 hours post-injury. The majority of the remaining patients were incapable of earlier weaning from bed-rest owing to fractures of an inferior limb or pelvis, spinal injury and disorders of consciousness due to head injuries.

**Table 1:** Clinical characteristics of patients successfully treated with nonoperative management for blunt abdominal trauma.

Characteristics	Patients successfully treated with NOM (n = 62)
Age (median, range)	37 (18–83)
Gender (male)	44 (70.9%)
Vital signs on arrival (median, range)	
Systolic ^1^BP (mmHg)	119 (60–202)
Heart rate (b.p.m)	97 (48–124)
^2^Shock	19 (30.6%)
^3^GCS	15 (3–15)
Injured organ	
Liver	28
Spleen	19
Kidney	15
High-grade Injury (^4^OIS IV-V)	27 (43.5%)
Extravasation on initial ^5^CT	19 (30.6%)
^6^ISS (median, range)	22 (4–57)
^7^PT (%) (median, range)	86.5 (27–128)
Fibrinogen (mg/dl) (median,range)	206.5 (52–570)
^8^NOM with TAE	16 (25.8%)
Days of second ^5^CT	7 (2–14)
(post injury) (median, range)	

^1^BP; blood pressure.

^2^Shock indicates patients whose systolic blood pressure was less than 90 mmHg or whose shock index (systolic pressure/heart rate) was < 1.0.

^3^GCS; Glasgow coma scale.

^4^OIS; organ injury scale.

^5^CT; computed tomography.

^6^ISS; injury severity score.

^7^PT; pro-thrombin activity.

^8^NOM with TAE; patients who were treated with nonoperative management with trans-catheter arterial embolization.

### Comparison of the clinical characteristics between the patients with and without new-onset PAs

As shown in Table 2, 7 out of 62 (11%) patients developed new-onset PAs after admission. The organs associated with the occurrence of PAs were as follows: the liver (n = 2), the spleen (n = 4), and the kidney (n = 1). There were no significant differences between the two groups with respect to the clinical characteristics (such as age, gender, vital signs on arrival, ratio of high-grade injury and the existence of extravasation on initial CT, PT-act, fibrinogen levels and ISS). Moreover, the ratio of patients who required TAE for the initial treatment was also not significantly different between the two groups. Both the bed-rest period and the ratio of early mobilization were not significantly different between the two groups. Lastly, there were no cases of delayed hemorrhage or death in either of the groups.

**Table 2:** Comparison of the clinical characteristics between the patients with and without new-onset of pseudoaneurysms.

	PA(+)		PA(-)		P-value
Age	39	(18–81)	37	(18–83)	0.74
Gender (male)	6	(86%)	38	(69%)	0.36
Vital signs on arrival					
Systolic ^1^BP (mmHg)	114	(60–130)	120	(60–202)	0.38
Heart rate (b.p.m)	90	(61–119)	97	(48–124)	0.99
^2^GCS	11	(3–15)	15	(3–15)	0.21
Inured organ					0.27
Liver	2	(29%)	26	(47%)	
Spleen	4	(57%)	15	(27%)	
Kidney	1	(14%)	14	(26%)	
High-grade injury (^3^OIS IV-V)	3	(43%)	24	(44%)	0.43
Extravasation on initial ^4^CT	3	(43%)	15	(27%)	0.40
^5^ISS	24	(4–57)	22	(4–45)	0.45
^6^PT (%)	92	(47–105)	85	(27–128)	0.43
Fibrinogen (mg/dl)	190	(78–570)	210	(52–343)	0.84
Bed-rest (days)	8	(2–27)	4	(1–41)	0.51
^7^Early mobilization	3	(43%)	16	(29%)	0.46

^1^BP; blood pressure.

^2^GCS; Glasgow coma scale.

^3^OIS; organ injury scale.

^4^CT; computed tomography.

^5^ISS; injury severity score.

^6^PT; pro-thrombin activity.

^7^Early mobilization; defined as mobilization from the bed within three days post-injury.

### Clinical characteristics and outcomes of the patients withPAs

All of the patients were diagnosed with PA by follow-up CT scan approximately 3 to 7 days after the injury and had no clinical symptoms associated with PAs such as abdominal pain, hematemesis, tarry stools and hematuria (Table 3).The median diameter of the PAs was 7.7 mm (range: 6.1 mm- 11.9 mm); the one patient who had a PA measuring more than 10 mm in diameter and a finding of grade III of splenic injury on initial CT received TAE immediately after follow-up CT, and no delayed bleeding was seen. All of the remaining six patients had PAs measuring less than 10 mm in diameter and were managed by observation only, without TAE or a restriction of activity. In all of these six patients, spontaneous disappearances of the PAs were observed on follow-up CT 3 to 26 days (median: 8 days) after the detection. None of the patients with PAs had any intra-abdominal hemorrhaging either during the hospital stay or after discharge; thus, none of the patients fell into NOM failure.

**Table 3:** Clinical characteristics and outcomes of the patients with PAs.

Day of detection (post injury) (median, range)	5	(3–7)
Diameter (mm) (median, range)	7.7	(2–11.9)
Larger 10mm in diameter	1	(14%)
Treatment		
^1^TAE	1	(14%)
observation	6	(86%)
Outcome of observation cases		
spontaneous disappearance	6	(100%)
Duration from detection to spontaneous disappearance (days)	8	(3–26)
Delayed hemorrhage	0	

^1^TAE; trans-catheter arterial embolization.

### Multivariable logistic regression analysis

The clinical characteristics such as age, ISS, PT-act, and fibrinogen levels were dichotomized at 55 years, 16, 70%, and 150 mg/ml, respectively, for the subsequent comparisons. A multivariable logistic regression model analysis, into which all of the clinical factors defined as risk factors for NOM failure by previous reports were entered, indicated that all of the factors (including early mobilization) were not found to be independent risk factors to the occurrence of PAs (Table 4).

**Table 4:** Results of the multiple logistic regression analysis of the occurrence of Pas.

	Odds ratio	95% confidence interval	*P*-value
Age>55 years	3.78	0.45–41.81	0.37
Male	3.04	0.31–81.00	0.40
Injured organ			
Liver	reference		
Spleen	2.76	0.35–30.89	0.34
Kidney	0.64	0.02–8.82	0.74
^1^PT<70%	1.46	0.04–35.30	0.82
Fibrinogen<150 mg/ml	1.11	0.03–25.63	0.95
High-grade injury (^2^OIS IV-V)	1.08	0.15–9.50	0.94
Extravasation	1.96	0.24–14.84	0.51
^3^ISS≥16	0.81	0.08–8.48	0.86
^4^Early mobilization	1.49	0.12–16.34	0.74

^1^PT; pro-thrombin activity.

^2^OIS; organ injury scale.

^3^ISS; injury severity score.

^4^Early mobilization; defined as mobilization from the bed within three days post-injury.

## Discussion

NOM has become the standard treatment for hemodynamically stable patients with blunt abdominal solid organ injuries, especially for those with low-grade injury scores. The success rate is reported to be between 78% and 98% [9,12,32–35] and it was 93% in this study, which is thought to be an acceptable result. Owing to expansion of the indications for NOM, more complications such as abscess formation, bile leaks, bilomas, urinomas, missed injuries, PAs and delayed hemorrhage have been reported [5,36,37]. Particularly, PAs gradually enlarge until they eventually rupture; therefore PAs have been recognized as a significant risk factor of delayed hemorrhage which may lead to NOM failure and fatality in some cases [38]. Therefore, we believe that the occurrence of posttraumatic PAs should be carefully monitored by follow-up CT for the patients managed with NOM, although there has been opposition to routine follow-up CT scanning. Cox et al. reported that only three out of 530 patients with blunt hepatic injury needed intervention based on the findings of the follow-up CT; furthermore, all of them revealed clinical signs suggestive of hepatic abnormality, such as tachycardia, abdominal pain and enzyme elevation [39]. Consequently, the authors concluded that routine follow-up CT scanning was unnecessary for patients with blunt hepatic injury managed by NOM when the patients had no clinical symptoms or signs. Hann et al. also reported that follow-up CT performed 48 to 72 hours after admission was not beneficial for patients with low-grade splenic injuries and without decreasing hematocrit levels [40]. In contrast, some authors have emphasized the efficiency of routine follow-up CT for detecting delayed onset PAs in patients with no symptoms, which would contribute to an early embolization before rupture and a decrease of NOM failure [12,26].The present study revealed that the occurrence rate of PAs after admission was as high as 11%, and 14% of those required additional interventions based on the findings of follow-up CT although the patients exhibited no clinical symptoms. Furthermore, more than 50% of those with PAs were in the low-grade injury group as classified by initial CT. Accordingly, we also recommend routine follow-up CT scanning regardless of the injury grade and the presence or absence of clinical symptoms and signs.

We recognize that a PA is a major risk factor for delayed hemorrhage, leading to NOM failure, however, we do not think that all of the PAs, irrespective of the diameter, are related to rupture. In the past case reports supporting the effectiveness of embolization for posttraumatic PAs, most of the patients complained of symptoms such as abdominal pain, melena, hematemesis, hematuria, etc [20–22,41,42]. We believe that these cases should be treated immediately, because these symptoms may suggest a threatened rupture or active bleeding from PAs. However, the majority of PAs, even in asymptomatic patients, have often been treated with prophylactic embolization because they have been implicated as the source of delayed rupture [43]. Some reports have emphasized the usefulness of prophylactic embolization for post-traumatic PA [44], however, a systematic review that analyzed the outcomes of posttraumatic PAs revealed no evidence to support or dispute the need for embolization of PAs [43].

In our study, six of the seven patients (86%) who had developed a PA that was less than 10 mm in diameter during the follow-up period were treated by observation only, without TAE, and no delayed hemorrhage was seen, while TAE was performed for one patient with posttraumatic PAs larger than 10 mm in diameter. Furthermore, spontaneous disappearances of the PAs were recognized by the follow-up CT in all of the six patients 3–26 days after the diagnosis. Muroya et al. also described that PAs of the spleen, ranging from 3.5 mm-25 mm in size, spontaneously occluded 2–10 days after detection. This demonstrated that small PAs could be treated without intervention, although how the size of PAs would affect treatment with TAE was not definitively established [26]. Crauford et al also described in their report that many small PAs had disappeared spontaneously without any intervention [23], and there are other cases reports about PAs that disappeared spontaneously [24,25], but these reports did not fully describe the PAs that underwent spontaneous resolution (e.g. the diameter, location, etc). Based on our experiences, the possibility of the spontaneous resolution of PAs could be expected when the diameter of the PA was less than 10 mm. The median duration from the diagnosis to spontaneous resolution of PAs was 8 days in the present study, which was similar to the average period described by previous reports (e.g., 5 days [26] and 12 days [23]). Consequently, those small PAs would be expected to spontaneously disappear within 1–2 weeks, and TAE for PAs could be withheld in this period unless the size of PAs increased. With the advances being made in CT technologies, the chance of detection of small-sized PAs will likely become more common, however, these small PAs would not be indicated for treatment [23,26].

Considering the risk of delayed hemorrhage, many trauma surgeons have forced patients with abdominal solid organ injuries to be kept on strict bed-rest for several days [5]. This is because patient movement or an unexpected fall is thought to cause disruption of a stable clot, which leads to delayed hemorrhage, although there is no definite evidence that this is the case. Even in the NOM practice guidelines published by the Eastern Association for the Surgery of Trauma (EAST) [1,2], issues about the duration and intensity of restricted activity are described as unanswered questions. Recently, some authors have revealed that a shorter length of hospitalization had not increased the occurrence of delayed hemorrhage, and had led to reduced costs and resource use [3,23], although there have been no reports describing the relationship between new-onset PAs and the duration of bed-rest. The present study is the first report to show that earlier mobilization was not significantly related to the occurrence of posttraumatic PAs. Since immobility on a bed is associated with the hazard of aspiration pneumonitis and pulmonary embolism from deep venous thrombosis [45], unnecessary bed-rest should be avoided.

## Study Limitations

There are several limitations in this study. First, this is a retrospective study with a small number of patients and only 19 out of 62 (31%) patients were mobile within 3 day after the injury. A prospective study with a larger number of patients and a high success rate of early mobilization is needed to confirm our results suggesting that PAs measuring less than 10 mm in diameter are expected to disappear spontaneously and that the occurrence of PAs is not related to early mobilization. Moreover, in this study, mobilization within three days after the injuries was defined as early mobilization; however, much earlier mobilization may be possible, especially in patients with a low injury score. An appropriate duration of immobilization should be prospectively investigated. The clinical characteristics of posttraumatic PAs, including potential for rupture distributing to delayed hemorrhage, would vary by the organs because of the difference of the tissue density and intra-parenchymal pressure. So, the indication size for TAE is may have been decided by the organs in which PAs occurred, although the difference of organs was not dependent factor of the PAs occurrence in this study. Furthermore, the probability of rupture of small-sized PAs less than 10mm in diameter is unknown, so conservative therapy should be performed in an environment that provides capabilities for monitoring, serial clinical examinations (such as those using enhanced CT), and an angiography or operating room available in case there is a sudden rupture of a PA.

## Conclusion

In conclusion, this study revealed the probability of spontaneous resolution of PAs when the diameter was smaller than 10 mm, and this is the first report which confirms that there is no significant relationship between the occurrence of posttraumatic PAs and early mobilization. Thus, unnecessary embolization for PAs and immobilization after trauma due to concerns about delayed hemorrhage following the occurrence of PAs should be avoided.
